# A new lever reduction technique for the surgical treatment of elderly patients with lumbar degenerative Spondylolisthesis

**DOI:** 10.1186/s12891-019-3028-8

**Published:** 2020-01-07

**Authors:** Chao Kong, Wei Wang, Xiangyu Li, Xiangyao Sun, Junzhe Ding, Shibao Lu

**Affiliations:** Department of Orthopedics, Beijing Xuanwu Hospital, Capital Medical University, Beijing, 100053 China

**Keywords:** Lumbar degenerative spondylolisthesis, Elderly, Reduction method, Lever reduction, Level of Evidence: IV.

## Abstract

**Background:**

Proper reduction method for Lumbar degenerative spondylolisthesis (LDS) is still controversial. The aim of this study was to determine the safety and effectiveness of lever reduction combined with traditional elevating-pull reduction technique for the treatment of elderly patients with LDS.

**Methods:**

From May 2015 to December 2017, 142 elderly patients (≥65 years) diagnosed with LDS were enrolled in this study with a mean follow-up of 25.42 ± 8.31 months. All patients were operated using lever reduction combined with traditional elevating-pull reduction technique. Patient age, sex, body mass index, bone mineral density, preoperative comorbidities, surgical duration, blood loss, and surgical complications were collected form patient charts. Clinical data as visual analog scale (VAS), Oswestry Disability Index (ODI), and 36-Item Short Form Health Survey (SF-36) were collected preoperatively, 1 month postoperatively, and at the final follow-up. Radiographic evaluation included slip percentage, slip angle (SA), lumbar lordosis (LL), and fusion status.

**Results:**

The clinical parameters of VAS_back_, VAS_leg_, ODI, and SF-36 had significantly improved at both follow-ups after surgery. A significant improvement was indicated for slippage reduction at both follow-ups, showing no significant correction loss after surgery. SA significantly increased after surgery and was well maintained at the final follow-up. LL was not affected by the surgery. At the final follow-up, complete fusion was obtained in 121 patients (85.2%) and partial fusion in 21 (14.8%). Revision surgery was performed for one patient. Screw loosening was observed in 3 (2.11%) cases. No nerve root injury or adjacent segment disease was observed.

**Conclusions:**

This new lever reduction combined with traditional elevating-pull reduction technique for the surgical treatment of elderly patients with LDS is both safe and effective. Satisfactory correction and fusion rates were achieved with acceptable correction loss and reduction-related complications.

## Background

Lumbar degenerative spondylolisthesis (LDS) is a common spinal degenerative disorder that can cause low back pain and radiculopathy if combined with lumbar stenosis [1–3]. According to the classification proposed by Meyerding [4], LDS often causes low-grade slippage (grades 1 and 2) due to an intact neural arch. For most patients with LDS, conservative treatments as physical therapy, medication, lifestyle management, and multidisciplinary pain management are effective [5, 6]. Surgical approaches are prompted in some refractory cases to achieve greater pain relief and functional improvement after mid-term follow-up [7, 8]. The aim of surgery includes decompression of neural encroachment and stabilization of the spinal column. Despite the large number of published studies, there is no consensus on the most appropriate surgical method [9, 10]. Most of the debate revolves around whether reduction should be performed and to which extent.

Many surgeons favor “in situ” fusion, which is the most common treatment method for LDS [11, 12]. However, in situ fusion is associated with the potential risks of pseudarthrosis and slippage progression, and is insufficient to restore lumbar lordosis [9]. To improve the fusion rates and restore sagittal alignment, instrumentation reduction is recommended for some patients. However, instrumentation reduction may cause screw loosening and loss of reduction, especially in elderly patients with osteoporosis [13, 14].

Traditional reduction methods mainly rely on distraction of the disc space and direct elevating-pull of the pedicle screws to obtain satisfactory reduction. But, in osteoporosis patients, poor bone quality may lead to inadequate bone-screw contact forces and, thus, more implant-related complications. Lian et al. [15, 16] reported three cases in which the pedicle screws in the slipped vertebra were pulled out during intraoperative reduction. However, it remains controversial whether reduction of elderly LDS patients with low bone mineral density (BMD) should be avoided [17].

To reduce the surgical related complications caused by reduction, we adopted a new lever reduction technique combined with traditional elevating-pull reduction technique during the reduction of elderly LDS patients in March 2014. Therefore, the aim of the present study was to evaluate the safety and effectiveness of this lever reduction technique for the treatment of elderly LDS patients.

## Methods

### Patient demographics

The study cohort prospectively included a total of 148 elderly patients (age, ≥65 years) diagnosed with LDS from May 2015 to December 2017 who met the following inclusion criteria: 1) a diagnosis of LDS and symptomatic lumbar spinal stenosis; 2) unresponsive to conservative treatments for more than 3 months; 3) instability of flexion and extension, as determined by X-ray; and 4) availability of at least 12 months of follow-up data. Patients with pars defects, hip disorders, previous spinal surgery or trauma, or incomplete data, were excluded from analysis.

Demographic and clinical data, including age, sex, body mass index, BMD, preoperative comorbidities, surgical duration, blood loss, and surgical complications were collected from electronic charts. The World Health Organization defines normal BMD as a T*-*score greater than − 1.0. Low bone mass or osteopenia is defined as − 1.0 to − 2.5, whereas T*-*scores equal to or less than − 2.5 indicate osteoporosis [18].

The visual analog scale (VAS), Oswestry Disability Index (ODI), and 36-Item Short Form Health Survey (SF-36) were used to measure outcomes. Clinical data were collected before surgery, 1 month postoperatively, and at the final follow-up. Preoperative lumbar radiographs (standing anteroposterior and lateral, flexion, and extension views), computed tomography, and magnetic resonance imaging were obtained from all patients.

### Radiological assessment

Plain radiographs were obtained preoperatively, 1 month postoperatively, and at the last follow-up. Preoperative and postoperative radiographic evaluation included slip percentage (SP), slip angle (SA), and lumbar lordosis (LL). Vertebral SP was measured as the percentage of slippage in relation to the lower vertebral body length, according to the Taillard technique [19] and graded according to the Meyerding classification [20]. SA was defined as the angle between the lower end plate of the slipped vertebrae and upper end plate of the lower vertebrae. Positive values were used to denote lordosis. LL was defined as the Cobb angle between the upper L1 end plate and upper S1 end plate. The reduction rate was calculated as: reduction rate (%) = (preoperative-postoperative SP) * 100/preoperative SP. All measurements were performed and repeated by two blinded observers.

Fusion was assessed on post-operative computed tomographic images obtained at the final follow-up according to the classification of Tan et al. [21] (Table 1). Pedicle screw loosening was viewed as any pedicular cortical breach or directional change [22].

**Table 1:** CT-based Classification of Spinal Fusion

Grades	Description	Criteria
Grade I	Complete fusion	Cortical union of the allograft at bone cranial and caudal ends and continuity of trabecular pattern between the autograft within the medullary canal of the allograft and the adjacent cranial and caudal vertebral bodies
Grade II	Partial fusion	Cortical union of the allograft to the endplates at each end however with partial or absent trabecular continuity between the medullary autograft and the adjacent vertebral body bone at one or either end.
Grade III	Unipolar pseudarthrosis	Cranial or caudal cortical nonunion of the allograft with associated central trabecular discontinuity
Grade IV	Bipolar pseudarthrosis	Both superior and inferior cortical nonunion with a complete lack of central trabecular continuity

### Surgical technique

**Fig. 1:**
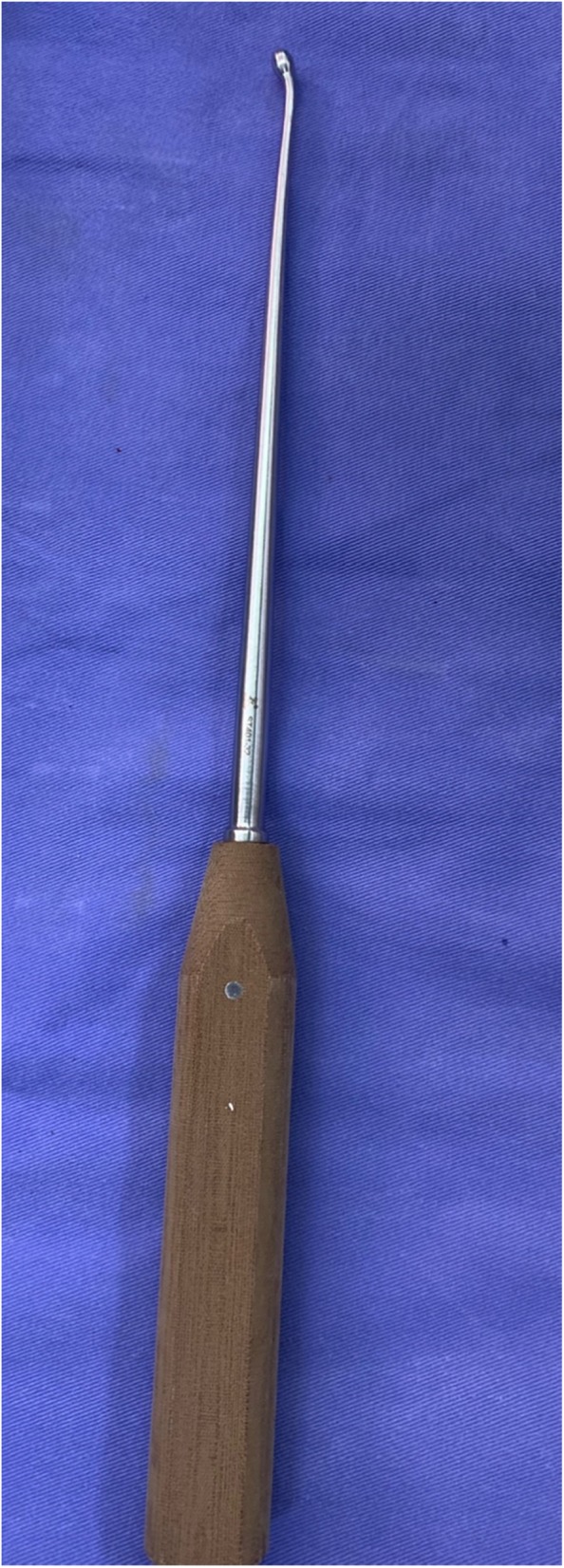
Illustration of an independently developed reduction facility

**Fig. 2:**
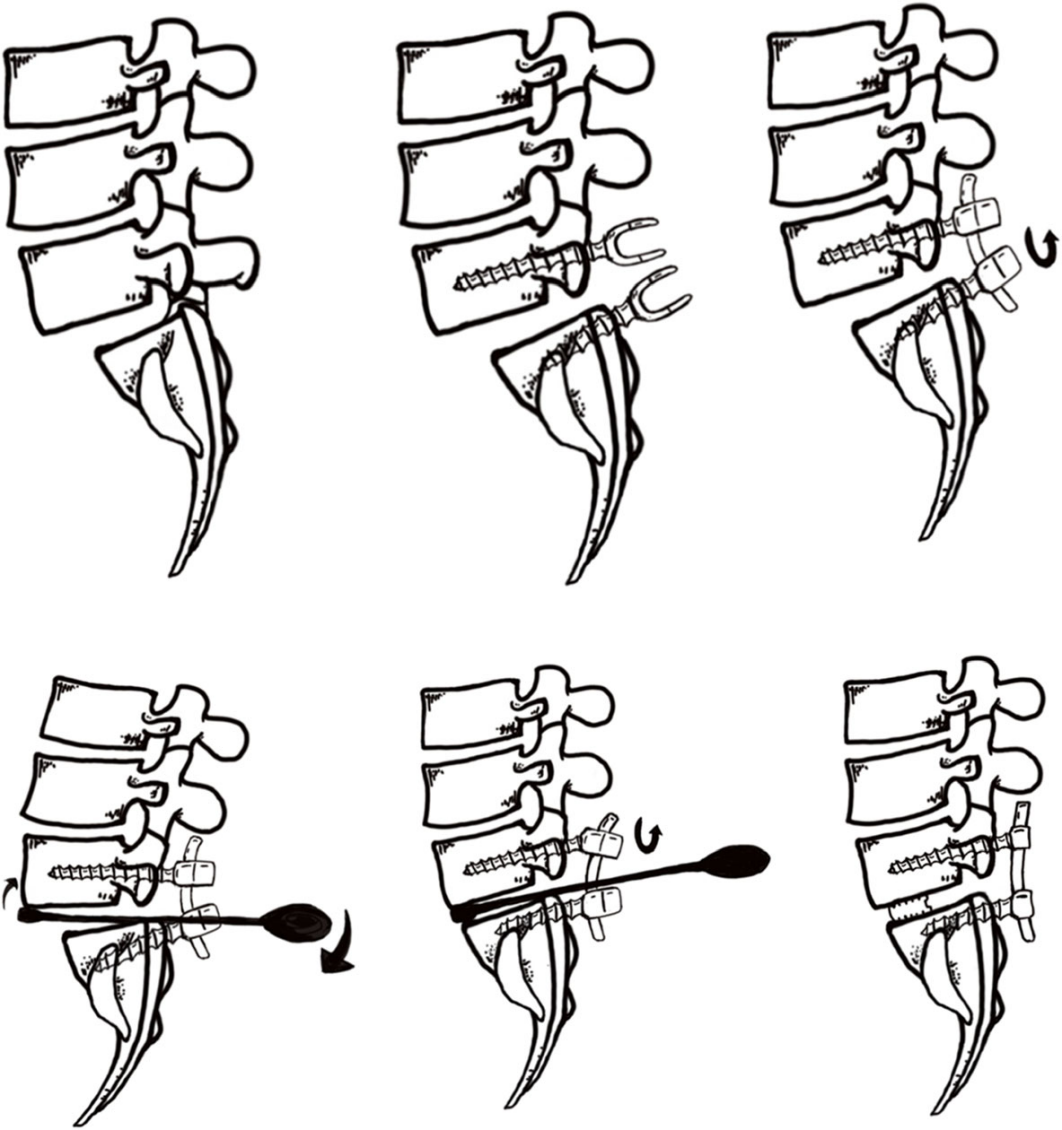
Reduction process of a slipped vertebrae. **a**, Forward slippage of L5; **b**, Pedicle screws were placed at both vertebra of the slipped levels; **c**, The nerve roots were decompressed before reduction. After removal of the disk tissues and endplate preparation, a rod was placed unilaterally and the pedicle screw of the lower vertebrae was locked; **d**, A lever repositioner was placed at the anterior rim of the slipped vertebrae under fluoroscopy; **e**, With the lower vertebrae as the lever fulcrum, force was applied to gradually pry the slipped vertebrae upward; **f**, The pedicle screws of the slipped vertebrae were locked. Then, an addition rod was placed and all screws were locked. The extent of slip reduction was verified with fluoroscopy. After reduction, the interspace was packed with autologous bone graft material and an appropriate cage filled with bone was inserted into the disc space

**Fig. 3:**
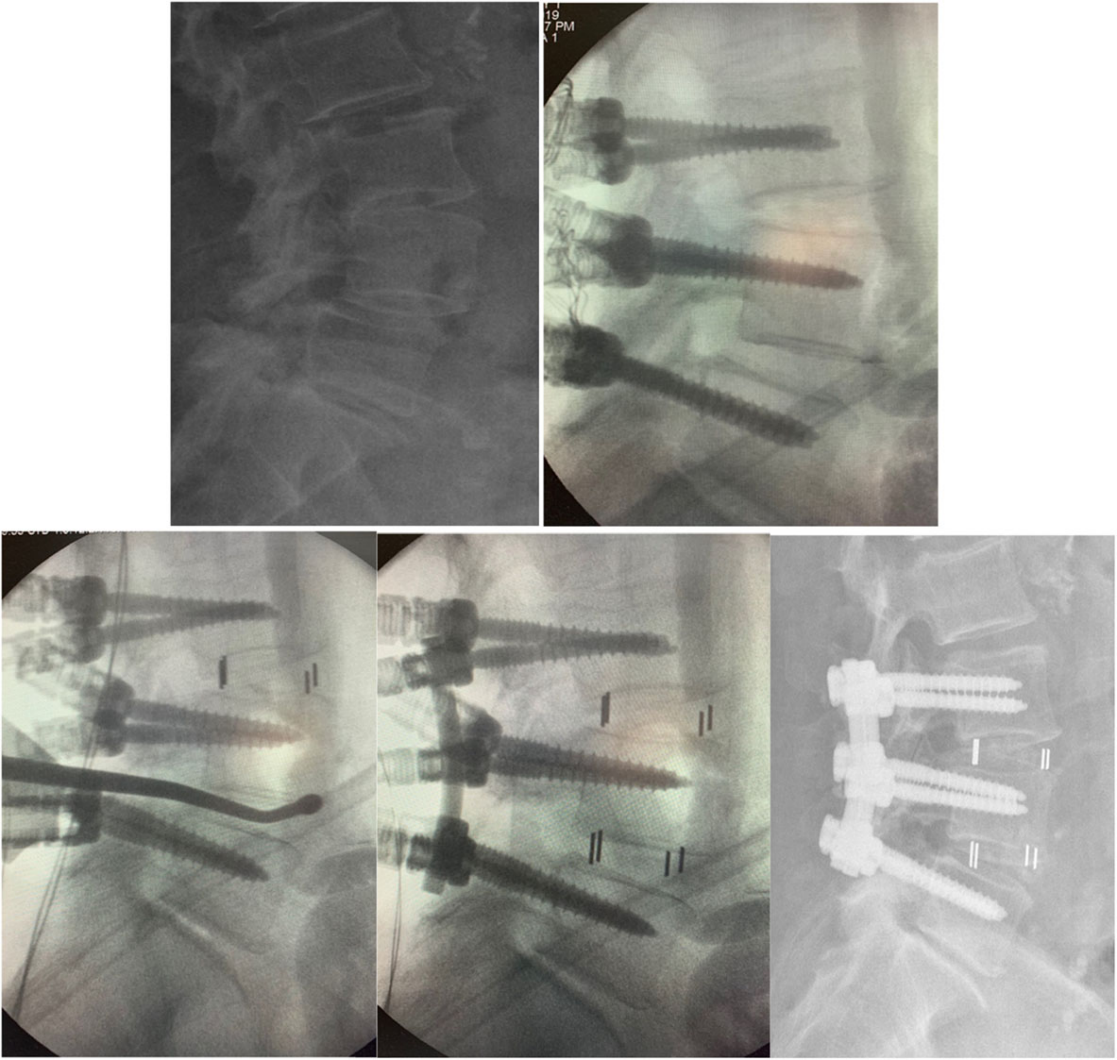
Female patient, 74 years old, diagnosed of lumbar degenerative spondylolisthesis and lumbar stenosis (L3/4 and L4/5). **a**, Forward slippage of L4; **b**, Pedicle screws were placed from L3 to L5; **c**, After decompression of both levels and cage insertion at L3/4, a lever repositioner was placed at the anterior rim of the slipped vertebrae under fluoroscopy; **d**, A rod was placed unilaterally and the pedicle screws of L3 and L5 were locked. After L4 was pried upward, the pedicle screw of L4 was locked and cage was then inserted; **e**, Postoperative X-ray showed satisfactory reduction of L4

All surgeries were performed through an open posterior midline approach. Before bony decompression, bilateral pedicle screws were placed. Decompression consisted of bilateral facetectomy and partial foraminotomy, including the hypertrophic ligament flavum. The disc space was opened and thoroughly cleaned with intradiscal drills and pituitary rongeurs. The cartilaginous endplates were cleaned with caution so as to not cause injury to the bone endplates. Bilateral nerve roots were liberated before reduction. An independently developed reduction facility was used during the surgery (Fig. 1). The reduction process consisted of five steps (Fig. 2): 1) placement of pedicle screws at both vertebra of the slipped levels; 2) decompression of the nerve roots before reduction (after removal of the disk tissues and endplate preparation, a rod was placed unilaterally and the pedicle screw of the lower vertebrae was locked); 3) placement of a lever repositioner at the anterior rim of the slipped vertebrae under fluoroscopy; 4) with the lower vertebrae as a lever fulcrum, force was applied to gradually pry the slipped vertebrae upward; and 5) during the lever reduction process, lock the pedicle screws of the slipped vertebrae. Then, an additional rod was placed and all screws were locked. The extent of slip reduction was verified with fluoroscopy (Fig. 3). After reduction, the interspace was packed with autologous bone graft material and an appropriate cage filled with bone was inserted into the disc space. During reduction, no distraction was used.

### Statistical analysis

All statistical analyses were performed using PASW Statistics for Windows, version 18.0 (SPSS Inc., Chicago, IL, USA). Continuous data are presented as the mean ± standard deviation (SD). The Kolmogorov–Smirnov test was used to determine whether the variables were normally distributed. One-way analysis of variance was used to identify differences among the clinical and radiological parameters at different time points. A probability (*p*) value of < 0.05 was considered statistically significant.

## Results

### Study population

A total of 142 elderly patients who underwent surgery in our institute for a diagnosis of LDS were followed for a mean of 25.42 ± 8.31 (range, 14–41) months. Six patients who lived in other cities and were unwilling to travel were lost to follow-up. The demographic data and intraoperative characteristics of all patients are shown in Table 2. The mean BMD was 0.821 ± 0.164 g/cm^2^. The T-scores > − 1.0 (normal BMD) were in 34 (23.9%) patients, between − 1 and − 2.5 (osteopenia) in 79 (55.6%), and less than − 2.5 (osteoporosis) in 29 (20.4%).

**Table 2:** Demographic Data and Intraoperative Characteristics of All Patients

Variables	Data
Age	72.8 ± 6.91
Gender(female/male)	104/38
Mean follow-up (month)	25.42 ± 8.31
Smoking (yes/no)	18/124
Alcohol (yes/no)	10/132
Body mass index (BMI)	26.12 ± 3.1
Bone mineral density (g/cm^2^)	0.821 ± 0.164
Number of comorbidities	
< 3	121
≥3	21
Meyerding grade, I/II	98/44
Surgical time (min)	183 ± 91.5
Blood loss (ml)	431 ± 426.7
Hospital stay (day)	15.62 ± 6.59

Data are presented as the mean ± SD

The distribution of slipped levels was: L2/3 in 2 cases, L3/4 in 18, L4/5 in 89, and L5/S1 in 38. Five patients had two slipped levels (L3/4 and L4/5 in 4 cases, L4/5 and L5/S1 in 1 case). According to the preoperative Meyerding classification, 98 patients had grade I (0% < SP < 25%) and 44 had grade II (25% < SP < 50%). Of all patients, 18 had lumbar degenerative scoliosis with an average Cobb angle of 17.6°. The distribution of fusion levels was: 1 segment (48, 33.8%), 2 segments (59, 41.5%), 3 segments (18, 12.7%), and > 3 segments (17, 12.0%). The average number of fused segments was 2.32.

### Clinical outcomes

Comparisons of clinical and radiological parameters at different time points are shown in Table 3. The clinical parameters of VAS_back_, VAS_leg_, ODI, and SF-36 had significantly improved at both follow-ups after surgery (*p* < 0.001). There were no statistical differences in clinical parameters from 1 month after surgery to the final follow-up (*p* > 0.05).

**Table 3:** Comparisons of Clinical and Radiological Parameters Preoperatively and Postoperatively

Parameters	Preoperatively	1 month postoperatively	Final follow-up	*p* value
VAS_back_	7.3 ± 2.1	1.8 ± 0.9	2.2 ± 1.5	< 0.001^a^
VAS_leg_	6.8 ± 1.9	1.2 ± 0.6	1.4 ± 1.1	< 0.001^a^
ODI (%)	63.1 ± 21.3	20.4 ± 9.1	15.6 ± 6.8	< 0.001^a^
SF-36(%)	44.3 ± 16.7	65.7 ± 17.4	71.3 ± 15.8	< 0.001^a^
SP (%)	18.31 ± 9.38	5.44 ± 2.66	6.39 ± 2.78	< 0.001^a^
SA (°)	5.89 ± 4.87	9.47 ± 4.08	9.18 ± 4.33	< 0.001^a^
LL (°)	44.06 ± 12.69	45.53 ± 11.16	44. 83 ± 11.24	0.1573
LL_SS_ (°)	45.47 ± 14.45	46.07 ± 13.32	45. 65 ± 13.89	0.6738

Data are presented as the mean ± SD. VAS_back_, visual analog scale for back pain; VAS_leg_, visual analog scale for leg pain; ODI, Oswestry Disability Index; SF-36, 36-Item Short Form Health Survey; SP, slip percentage; SA, slip angle, LL, lumbar lordosis; LL_SS_, lumbar lordosis in patients with single segment fused. ^a^ means preoperative data was different from the data at 1 month postoperatively

### Radiological outcomes

Slippage reduction had significantly improved at 1 month after surgery (*p* < 0.001) (Table 3). Slip correction was 70.29% immediately after surgery. There was no significant difference in the mean SP at 1 month after surgery vs. the final follow-up (*p* = 0.452), demonstrating no significant correction loss after surgery. SA significantly increased after surgery and was well maintained at the final follow-up (Table 3). LL, on the other hand, was not affected by surgery. To minimize the effect of fused segments on LL, patients with a single fused segment were included for analysis, but there was no significant difference between pre-and post-operative LL (Table 3). No significant correction loss was observed in SA and LL. At the final follow-up, complete fusion (grade I) was achieved in 121 patients (85.2%) and partial fusion (grade I I) in 21 (14.8%).

### Complications

Revision surgery was performed in one patient because of cage malposition during hospitalization. Screw loosening was observed in 3 (2.11%) cases (one at 3 months after surgery and the other two at 6 months), all of which occurred at the S1 vertebrae. None of these patients complained of back or leg pain and all had achieved partial fusion at the final follow-up. Two patients developed deep wound infections and underwent debridement without removing the implant. No nerve root injury was observed during reduction. Deep vein thrombosis occurred in one patient, while pneumonia was observed in two. No adjacent segment disease was observed at the final follow-up.

## Discussion

Since first described by Jenkins et al. [23] in 1936, surgical reduction for the treatment of lumbar spondylolisthesis has remained controversial [24, 25]. In Kawakami’s study, severe low back pain and lower recovery rate were observed in patients after in situ fusion compared with those in patients with reduction [24]. Two comparative studies on the clinical results of patients with and without reduction failed to find any significant difference [26, 27]. However, both studies included relatively small numbers of patients and the heterogeneity made it impossible to draw a firm conclusion. Although slip reduction remains controversial, restoration of segmental alignment and sagittal balance is still appealing and may be beneficial to limit degeneration of the adjacent segment degeneration in the long-term follow-up [24].

Multiple techniques have been described for the reduction of spondylolisthesis. For example, Magerl et al. [28] used external transpedicular fixation to provide distraction and compression forces in the reduction process. But, there was a high rate of pin site complications requiring multiple surgeries, thus this technique is seldom used. Ilharreborde et al. [29] applied a cantilever maneuver to obtain reduction and create lordosis at the spondylolisthetic level and Ruf et al. [30] proposed the temporary use of transpedicular reduction screws as a means to combine distraction and pulling strength during reduction. Other reduction methods, including traction, transsacral interbody fusion technique, and minimally invasive lumbar interbody fusion, have also been used [31–33].

The techniques mentioned above meet two requirements: distraction of the disc space and pulling of the slipped vertebrae. Theoretically, distraction and pulling strength via pedicle screws required adequate contact force between the instrumentation and bone. But, in elderly patients with LDS, poor bone quality may cause greater risks of instrumentation failure [15, 16].

**Fig. 4:**
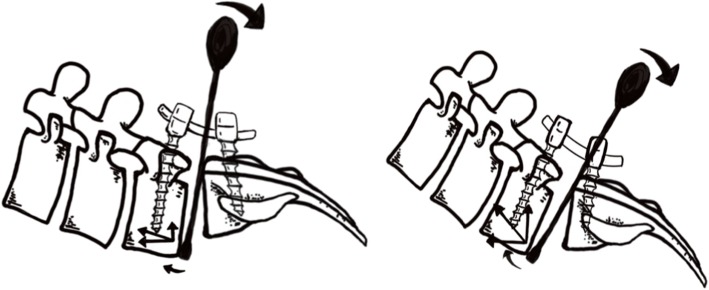
**a** The lever repositioner is placed more vertical leading to small decomposed force in the vertical direction (pulling strength). **b** The lever repositioner is placed more horizontal leading to large decomposed force in the vertical direction

To minimize the risks of implant-related complications during reduction, we adopted a new lever reduction technique combined with transforaminal lumbar interbody fusion for the surgical treatment of elderly LDS patients. After removing the disc and decompression of the nerve roots, a lever reduction assembly was placed at the anterior rim of the slipped vertebrae. Using the lower vertebrae as the lever fulcrum, force was applied to gradually pry the slipped vertebrae up and then lock the pedicle screws of the slipped vertebrae. During the reduction process, the force applied to the lever repositioner could be decomposed to horizontal and vertical stress, which served as distraction and pulling force, respectively. Since the pulling force is essential for reduction, the direction of the force applied to the lever repositioner will affect reduction (Fig. 4). Furthermore, lumbar lordosis, slip angle, slip degree, and disc space may affect the correction rate.

The reason for choosing the anterior rim as the prying point is based on the anatomic and biomechanical characteristics of the lumbar endplates. Peripheral regions have increased endplate thickness and bone density, as compared to the center of the endplate [34, 35]. A study by Liu et al. [36] found that the strength and stiffness was greater by 17% in the outer regions, as compared to the inner regions of the lumbar endplate. Although the anterior rim can provide reduction forces, care must be taken and the whole process should be completed under fluoroscopy. No intraoperative screw loosening was observed even though injury to the endplate could not be avoided.

In the current study, the average age of patients was 72.8 ± 6.91 years and 76% had osteopenia or osteoporosis. The complication rate in these elderly patients is relatively high. In a prospective study [15], pedicle screws were pulled out intraoperatively in two elderly patients (5.6%) using traditional reduction method and the complication rate was 22.2%. Another study reported a correction rate of 30.1% and a fusion rate of 64% on CT-scan for patients with low-grade spondylolisthesis after 1 year’s follow-up [22]. Okuda et al. reported a complication rate of 16% and a correction rate of 58.3% in elderly patients with a nonunion rate of 4% [37].

Compared with patients undergoing traditional reduction method, patients in this study had lower complication rate. Though most patients had poor bone quality, the clinical outcomes were satisfactory and a mean correction rate of 70.29% was achieved using this technique. No intraoperative screw loosening or other implant failure was observed, and all patients achieved complete or partial fusion at a mean follow-up of 25.42 ± 8.31 months.

The correction of segmental kyphosis and spinal alignment is also very important. The mean slip angle was 5.89 ± 4.87°, which increased to 9.47 ± 4.08° at 1 month after surgery and 9.18 ± 4.33° at the final follow-up. LL, on the other hand, was not affected by surgery. To minimize the effect of fused segments on LL, patients with a single fused segment were included and we also found no significant difference between pre-and post-operative LL. The correction loss was relatively small and resulted no statistical difference. With this technique, restoration of alignment is achieved via reduction of slippage and insertion of an intervertebral cage to avoid the potential risks of nerve root compression and screw loosening due to posterior compression.

One of the benefits of reduction is a greater fusion rate. In this study, all patients achieved complete or partial fusion at the final follow-up. Many studies have used plain radiographs to assess fusion integrity, including one that classified fusion as motion of less than 5° on dynamic lateral radiographs [38]. As noted by Boden et al. [39], the presence of residual segmental motion generally suggests non-union, although a lack of motion does not necessarily imply spinal fusion. Other authors have also suggested that the ability to demonstrate spinal fusion is limited on plain radiographs [40, 41]. To better evaluate the fusion rate, we used the fusion classification proposed by Tan et al. [21] on CT scan, which reflected the actual fusion status after surgery.

This study had some limitations. First, this is a prospective study with small number of patients and relatively short follow-up time. Secondly, this is not a comparative study lacking a control group with decompression and fusion without reduction. Hence, future studies are needed to compare this technique with in situ fusion and other reduction techniques.

## Conclusion

This new lever reduction technique combined with traditional elevating-pull reduction technique for the reduction of elderly patients with LDS is safe and effective. Satisfactory correction and fusion rates were achieved with acceptable correction loss and reduction-related complications. Further comparative studies between this technique and other reduction methods are needed in the future.

## Data Availability

The datasets used and/or analyzed during the current study are stored in our hospital and are available from the corresponding author on reasonable resquest.

## Abbreviations

LDS: Lumbar degenerative spondylolisthesis
LL: Lumbar lordosis
ODI: Oswestry Disability Index
SA: Slip angle
SF-36: 36-Item Short Form Health Survey
SP: Slip percentage
TLIF: Transforaminal lumbar interbody fusion
VAS: Visual analog scale
